# SToRytelliing to Improve Disease outcomes in Gout (STRIDE-GO) in African American veterans with gout: a trial study protocol

**DOI:** 10.1186/s13063-021-05847-9

**Published:** 2021-12-04

**Authors:** Jasvinder A. Singh

**Affiliations:** 1 Medicine Service, VA Medical Center, 510, 20th street South, FOT 805B, Birmingham, AL 35233 USA; 2 Department of Medicine at School of Medicine, University of Alabama at Birmingham, 1720 Second Ave. South, Birmingham, AL 35294-0022 USA; 3 Division of Epidemiology at School of Public Health, University of Alabama at Birmingham, 1720 Second Ave. South, Birmingham, AL 35294-0022 USA

**Keywords:** Gout, Storytelling intervention, Behavioral intervention, Randomized trial, African American, Racial/ethnic minorities

## Abstract

**Objective:**

Medication adherence in gout is suboptimal, and the lack of effective interventions to address it presents a huge challenge. Medication adherence and gout outcomes are worse in racial/ethnic minorities. The objective of this paper was to provide the details of the study protocol for randomized, controlled trial (RCT) in African Americans (AAs) with gout that will test the effectiveness of a culturally appropriate gout storytelling intervention.

**Methods:**

The SToRytelliing to Improve Disease outcomes in Gout (STRIDE-GO) study will be a 12-month, multicenter, open-label RCT that will assess the effect of a culturally appropriate gout storytelling in at least 300 AA veterans with gout. Participants will be randomized to gout-storytelling intervention vs. a stress reduction video in a 1:1 ratio. The primary outcome is urate-lowering therapy (ULT) adherence measured with MEMSCap™, an electronic monitoring system (efficacy, 6 months; sustenance of efficacy, 12 months). Secondary outcomes include gout flares, serum urate (SU), gout-specific health-related quality of life [HRQOL], self-reported ULT adherence, patient satisfaction with treatment, and patient understanding of the intervention. AA veterans with gout who met the 1977 Preliminary American College of Rheumatology (ACR) classification criteria for gout, currently prescribed an oral ULT medication (allopurinol or febuxostat) for at least 6 months, and not using a pillbox to redistribute their medications, will be invited to an in-person study visit. After the study coordinators obtain informed consent, and ensure that participants meet the inclusion criteria, the eligible participants will be provided with their current ULT in a MEMSCap™ bottle for the 1-month run-in period and asked to return to the clinic in 1 month. ULT adherence with MEMSCap™ will be recorded at a 1-month return visit. Interested participants will complete the baseline assessments, randomized using the computerized system to either gout-storytelling intervention or a stress reduction intervention video arm and watch the respective video in-clinic. Patients will be interviewed on the phone at 2 and 4 months regarding the viewing of the videos at home at each time. Participants will be assessed in-clinic at 3, 6, 9, and 12 months; MEMSCap™ data and patient surveys will be captured at each visit. For any missed visit, assessments will be completed on the phone and MEMSCap™ data captured at the next in-clinic visit.

**Discussion:**

The study will assess the efficacy of a behavioral intervention to improve ULT adherence in minority populations with gout.

**Trial registration:**

ClinicalTrials.gov NCT 02741700. Registered on 14 September 2018

**Supplementary Information:**

The online version contains supplementary material available at 10.1186/s13063-021-05847-9.

## Introduction

Medication non-adherence, i.e., not taking medications as prescribed, costs over $100 billion a year in excess hospitalizations in the USA [1]. The problem is worse in patients with gout, with only 37% of gout patients taking 80% of their prescribed medication in the first 12 months of the treatment [2]. Gout is the most common inflammatory arthritis. It affects 8.3 million Americans [3] and 5% of veterans [4] Its prevalence is increasing [3]. Multiple patient- and system-related barriers contribute to medication non-adherence [5, 6]. In a Cochrane systematic review, only 18 of 58 medication adherence interventions led to improved outcomes in chronic disease [7]. The successful interventions incorporated self-efficacy or peer group, i.e., were more patient-centered. Thus, more efficacious, feasible, low-cost behavioral interventions are needed to address non-adherence in chronic disease management, especially in gout.

Compared to Whites, Blacks or African Americans (AAs) have higher gout prevalence and incidence [3, 8] and worse outcomes, including higher serum urate (SU) and higher rate of emergency room visits or hospitalizations for gout [9]. Specifically, AAs are more non-adherent with urate-lowering therapy (ULT) [10] and lower rates of treatment with allopurinol, the most commonly used ULT [11]. A higher ULT non-adherence in AAs is related to patient knowledge gaps, perceptions, and perceived barriers to gout treatment [12]. Poor gout outcomes are also partially attributable to higher rates of hypertension [8], obesity, diabetes, and renal failure in AAs compared to Whites [13]. Thus, gout leads to a disproportionately higher disease burden in AAs compared to Whites.

A recent 6-month study using a patient storytelling intervention about their experience with the disease and its treatment showed improved hypertension control in AAs with hypertension [14]. The hypertension study represented a model for chronic asymptomatic diseases. Gout represents a model for chronic, intermittently symptomatic diseases, due to its well-elucidated biochemical abnormality, pathophysiology, treatment, and outcomes, all related through a single factor, SU. Because gout is associated with symptoms, we anticipate that storytelling may impact adherence in a manner that is different from patients with hypertension [14]. Gout presents a relatively simple model in which the gold standard surrogate for disease outcomes, SU, is primarily affected by ULT medication adherence [15, 16]. Therefore, we designed a 12-month, multi-center, randomized controlled trial (RCT) to assess the effect of a culturally appropriate gout storytelling in AA veterans with gout. This manuscript provides the protocol for this randomized trial.

The main objective of the randomized study is to assess the efficacy of a culturally appropriate gout storytelling in AA veterans with gout. We hypothesize that gout storytelling would improve ULT adherence more than the control intervention in AA veterans with gout.

## Methods

### Study objective

**Fig. 1:**
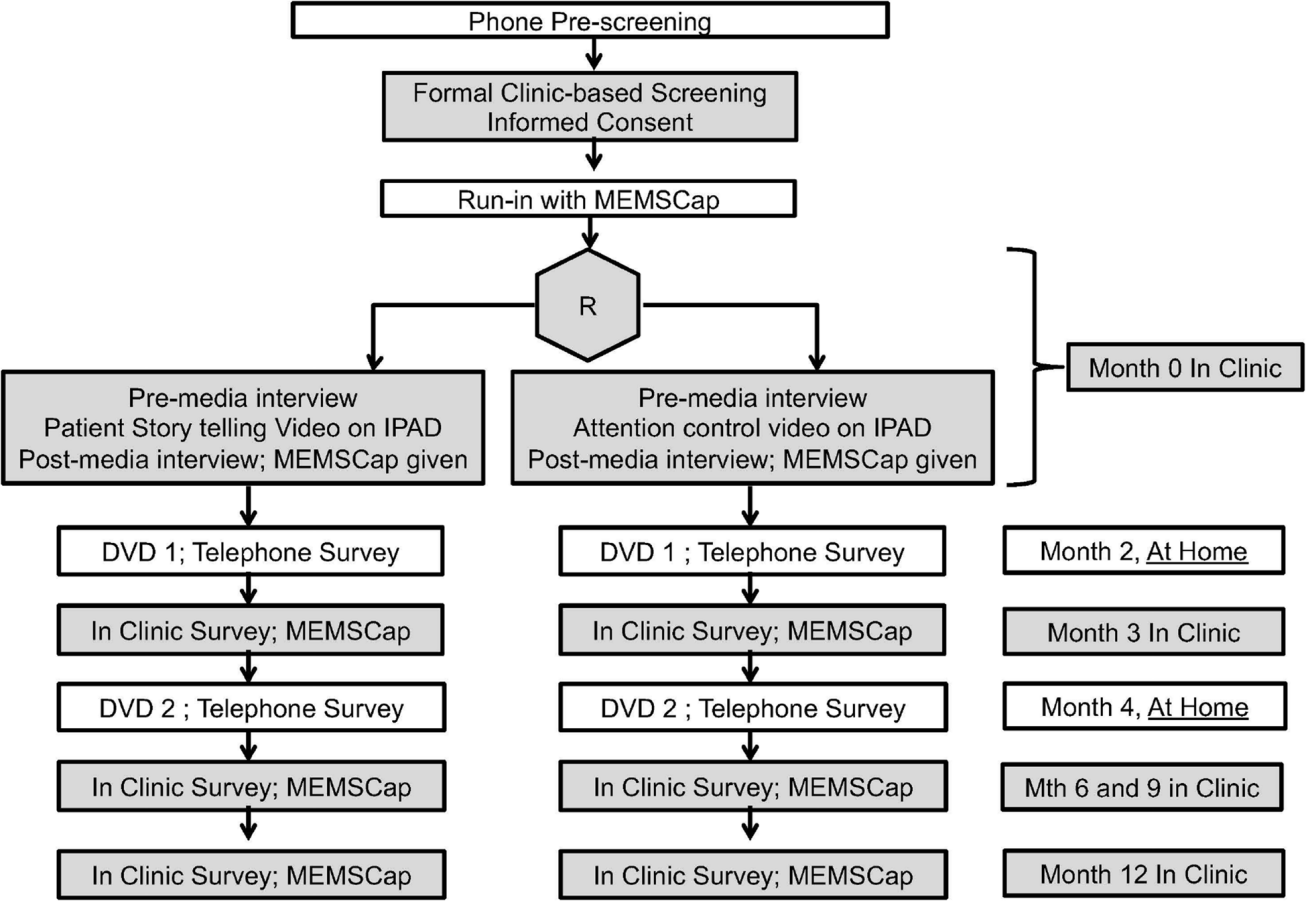
Study flow diagram. R, randomization. Study visits to be completed at home are shown without shading. Study visits to be completed in the clinic are shown with gray shading. Phone pre-screening and 2- and 4-month at-home telephone surveys will be done by the study coordinator on the phone at the pre-scheduled time with the patient. DVD1 and DVD2 will be provided to the patient per the patient preference at the preceding visit, or mailed to the patient and the receipt confirmed, prior to the call. All in-clinic surveys are patient self-administered outcome assessments completed directly on an IPAD by the patient during the in-clinic visits. In case a patient misses a scheduled study visit, they will be rescheduled for a study visit in the next 1–2 weeks. If that fails, or is not possible, a phone interview is done by the coordinator with the patient to complete all patient surveys, and the MEMSCap is read at the next in-clinic visit

The primary objective of this 1-year study is to assess the efficacy of a novel gout storytelling in patient’s own voice on ULT adherence in gout, assessed by an objective measure of ULT adherence assessed with MEMSCap™ electronic bottlecap monitoring at 6 months (primary trial end point) (Fig. 1). Secondary objectives are to assess patient outcomes including gout flare rate, patient satisfaction, target SU < 6 mg/dl achievement, health-related quality of life (HRQOL), self-reported ULT adherence, and patient understandability of the intervention.

### Study overview

Patients in the storytelling intervention group will view the designed storytelling video, and the usual care group will view a presentation on stress management for the same time duration (attention control), on an IPAD/touchpad in the clinic during the baseline visit. Mailed DVDs to the enrolled patients (or in-person at previous visits, per patient preference) at 2 and 4 months with additional storytelling clips will reinforce messages for behavioral change received during the baseline visit through storytelling videos; mailed DVDs to the attention control group will provide information on stress management. We will use the Medication Event Monitoring System Cap (MEMSCap™; Aprex Corp., Fremont, CA), a medication bottle cap with a microprocessor that records the occurrence and date and time whenever a patient opens a vial using integrated microcircuits*,* as a measure of the primary outcome of ULT adherence [17]. It will be dispensed at the baseline visit, and data downloaded at each in-person visit. Patients will receive the first 90-day supply in special bottles with MEMSCap™ and be trained at the initial visit regarding its importance and to bring it to the 3-, 6-, 9-, and 12-month clinic visits; opening/closing of the bottle cap will be demonstrated to participants.

### Study population, study sites, randomization, and ClinicalTrials.gov registration

We will conduct a multicenter, parallel 2-arm, 12-month open-label RCT comparing a culturally appropriate gout-storytelling intervention to a control intervention (stress reduction) in AA veterans with gout. We will recruit participants at Birmingham, Philadelphia, and St. Louis Veterans Affairs (VA) Medical Centers (VAMCs).

### Specific aims

Our specific aims (SAs) are to assess the efficacy of a gout storytelling intervention for the following:

- SA1: Improving ULT adherence, directly measured by using MEMSCap™ at 3, 6, 9 months (assess the intervention’s effect), and 12 months (assess the durability of effect)
- SA2: Improving gout flare rate, patient satisfaction, target SU < 6 mg/dl achievement, health-related quality of life (HRQOL), self-reported ULT adherence, and patient understandability of the intervention, as important gout outcomes.

### Intervention platform

The intervention will be delivered in a series of three videos in the proposed RCT (details below) at baseline using an IPAD and at 2- and 4-month DVDs sent via mail or provided in-person to the participant at the baseline and follow-up visits, per patient preference. Each video will either have the gout-storytelling intervention with patient narrated stories and “learn more” gout material in a patient’s own voice or the stress management video. The duration of the videos is the same (15–20 min). DVDs will have new stories from our storytelling stars, based on patient preference for messages (diet, medication, disease impact), but will be like those presented at the baseline visit.

Parallel study protocols for the intervention and comparison groups have been designed. Specifically, the contact with research personnel (including rapport building), duration of the intervention, mailings and phone calls, and the assessment schedules will be identical in the intervention versus attention control arms (Fig. 1).

### Study sites, personnel training, Institutional Review Board (IRB) approval, and trial registration

Birmingham, Philadelphia, and St. Louis VAMCs are the three study sites. After the approval for our study from each IRB, experienced research associates will be responsible for patient recruitment, informed consent, and randomization procedures. Trained study research assistants at each site will be supervised by the respective site PI. The study is registered on ClinicalTrials.gov website (NCT 02741700).

### Study advertisement for participation

We will advertise our study in three ways, like our previous studies: (1) via IRB-approved mailed letters to patients with gout who preliminarily meet the study eligibility criteria based on the VA pharmacy, administrative, and clinical database records and (2) through emails to the primary care physicians at regular intervals, every 2–3 months.

### Identification of the study population and pre-screening using pharmacy records and a phone call

Our study population is AA veterans with gout with low adherence, defined as an average ULT MPR < 0.80 (= #days of outpatient days’ ULT supply used/#days’ supply), based on the immediately prior period of 180 days (low adherers). Veterans will be identified with the help of the Automated Data Package Application Coordinator (ADPAC) at each facility. Using the VISTA pharmacy query, the research assistant at each VA site will obtain an automated monthly query in VISTA pharmacy records to capture lists of veterans at each VA site every week who filled a recent ULT (allopurinol or febuxostat) prescription, along with the dates of ULT refills in the last 6–9 months and days’ supply, to identify potentially eligible patients. Baseline adherence assessment using databases is different than primary outcome assessment with MEMSCAP™ due to feasibility. Knowing that the race/ethnicity variable is not recorded on all and sometimes recorded incorrectly in VA databases, we will preliminarily ascertain it from the patient’s inpatient and outpatient CPRS medical record and confirm it during phone pre-screening. The research assistant at each site will call and pre-screen eligible veterans for inclusion/exclusion criteria using the pre-screening questionnaire. Those that pass the pre-screen will be invited to come for a study screening and enrollment visit.

### Subject eligibility criteria for the STRIDE-GO study

The inclusion criteria are as follows: (1) AA veterans (*self-identified race will be the gold standard; bi- and multi-racial included*) aged 18 years or above with a diagnosis of gout (1 inpatient or ≥ 2 outpatient International Classification of Diseases, ninth revision, clinical modification [ICD-9-CM] codes 274.x or 274.xx) [4]; (2) meet the 1977 Preliminary American College of Rheumatology (ACR) classification criteria for gout [18]; (3) currently prescribed and filled oral ULT medication prescription (allopurinol or febuxostat) for at least 6 months; (4) ULT MPR < 0.80 for the preceding 6 months excluding the most recent filled ULT prescription for which the period of prescription fill (90, 30, or 60 days) has not been completed (the most common VA prescription is 90 days—see the “Protocol modifications” section); and (5) able to provide informed consent.

The exclusion criteria are as follows: (1) ULT MPR ≥ 0.80 (see the “Protocol modifications” section) and (2) patients who must redistribute daily pill into the pillbox.

The drop-out criteria are as follows: (1) patient refusal to continue to participate in the trial and (2) patient starting the use of a pillbox for the ULT.

### Subject screening, in-clinic enrollment, and baseline study assessments

For the in-clinic screening and baseline visit, patients will meet the research associate at their regular clinic location and, once their scheduled clinical visit ends, walk to a private area for more detailed screening and enrollment. Once enrolled in the study, study participants will meet the research associate at the study clinic location, if they are coming for the study visit only. Study participants will provide informed consent and HIPPA authorization. The site research associate will screen the veterans for study eligibility criteria, including the preliminary ACR gout classification criteria. For those who meet all the study inclusion criteria, the site PI will confirm eligibility for enrollment in the study.

Patients will then complete the baseline patient assessments including the following: (1) demographics—age, gender, income (covariates), and marital status; (2) gout duration, baseline frequency of gout flares requiring treatment, and baseline patient satisfaction with ULT treatment; (3) baseline gout-specific HRQOL assessment using the gout assessment questionnaire (GAQ); (4) alcohol use and body mass index (BMI) (covariates; alcohol and higher BMI are associated with a higher risk of incident gout, gout flares, and higher SU level [3, 19–21]); (5) blood draw for baseline SU; and (6) ULT non-adherence on the self-reported questionnaire by Voils et al. [22]. Additional information will be obtained including contact information, best time to contact, and email address. Veterans will be provided with a $25 remuneration for completing the study assessments at the baseline visit. Patients without DVD players will be provided with a DVD player to watch the DVDs at home at 2 and 4 months.

### Randomization and allocation to treatment and allocation concealment

Once patients complete all baseline assessments, they will be randomized into one of the two groups, gout storytelling versus control intervention. Randomization will be based upon a permuted variable block design. An online computerized simple randomization scheme will be programmed by a study biostatistician for each VA site with redundant systems established to avoid interruption during periods such as server upgrades and maintenance, available through a secure Internet link. This ensures that the allocation is concealed from the study assessors and the stud PI.

Due to the nature of interventions, patients will be aware of the group assignments. The two treatment groups are as follows:

- Group 1: Storytelling intervention
- Group 2: Usual care (attention control)

The intervention group will view the storytelling video modules in entirety at the baseline visit (in-person) and provided DVDs to view study month 2 (by mail/previous visit) and study month 4 (by mail/previous visit). Storytelling in African American veterans’ own voices will focus on improving ULT adherence, along with patient-narrated video segments about gout and its treatment under “Learn More,” by adapting a pre-tested power-point slide presentation narrated by a veteran with gout. The intervention group will also get a printed copy of the stories and the power-point presentation in the “Learn More” section at baseline. Each intervention installment will present new stories and Learn More gout content. Participants will be introduced to the MEMSCap™ and trained during their initial visit by research assistants.

The usual care comparison group will receive the attention identical to the intervention condition (attention control), aside from not including the gout storytelling modules. The usual care group will view a stress management video, based on the content adapted from the Centers for Disease Control in a power-point presentation and narrated by the same veteran, who narrated the gout power-point presentation. The video segments will be of the same length as in the intervention group.

### Patient recruitment and retention

We will stay connected with the study participants during the follow-up with mailed postcards and phone call reminders. We will mail study newsletters featuring images and quotes from consenting participants, after appropriate permissions from each site’s IRB. Study retention rates in the previous studies conducted by the PI that included minorities have exceeded 80% [23, 24]. Our current protocol allows for a dropout of similar magnitude, as the worst-case scenario. However, based on our experience and expertise, we expect the dropout rate to be lower.

### Study procedures at follow-up visits and data collection tools, including the 12-month blood draw

Follow-up assessments will be done at 3, 6, and 9 months after the baseline visit lasting 30 min in-person, 1 month via phone lasting 15 min and at 12 months lasting 1 h in person. One-month follow-up will be done via a telephone-administered survey at the patient’s convenience in their home. The 3-, 6-, and 9-month visits will be in-person, and MEMSCap™ data will be downloaded and other outcomes (gout flares, GAQ, and self-reported medication adherence) captured at each visit (Table 1). The schedule of collection of various outcomes is shown in Table 2. Post-card (and emails, when applicable) will be mailed 1 week and phone call made 2 days prior to the follow-up to remind the patient of the follow-up assessments. To minimize patient responder burden, veterans are only completing assessments related to primary and select secondary study outcomes (including a 4-item gout flare questionnaire) at each study follow-up visit.

**Table 1:** Summary of primary and secondary outcomes and outcome measures to achieve SA1 and SA2

	Description	Clinically meaningful change
**Primary outcome/follow-up time of assessment (SA1)**		
*6-month ULT medication* adherence measured using MEMSCap™*/3, 6, 9, and 12 months	Medication adherence to ULT measured using MEMSCap™ [25]	Absolute difference of 6% between the groups representing a medium effect size of 0.40 (a smaller difference is unlikely to be meaningful)
**Secondary outcomes (SA2)**		
*Number of gout flares/3, 6, 9, and 12 months*	Number of gout flares in the last 1 and 2 months Current flare: 4-item patient-reported assessment of gout flare [26]	20% fewer patients with gout flares needing treatment (absolute difference)
*Patient Satisfaction with Medications Questionnaire (SATMED-Q)/6 and 12 months*	17-item patient-reported with six dimensions [27]	Total score: 5.9 to 13.4 points SATMED-Q domain scores: 5.9 to 20.6, with most estimates close to 10 [28]
*Serum urate < 6 mg/dl/12 months*	Serum urate standard biochemical assay [29]	20% more patients achieving target serum urate < 6 mg/dl (absolute difference)
*Patient Education Materials Assessment Tool for Audiovisual Materials (PEMAT-A/V)/2 and 4 months*	Understandability (16 items), accountability (4 items), and potential impact of various messages on change in behavior, including ULT adherence [30]	No defined threshold
*Gout-Specific Health-Related Quality of Life (HRQOL) on Gout Impact Scale (GIS) of the Gout Assessment Questionnaire (GAQ)/3, 6, 9, and 12 months*	A validated measure of specific impact of gout on HRQOL [23]; 22 items (0–100 scale) that constitute 5 subscales	A clinically important difference of the GIS is between 5 and 8 points on each GIS subscale [31]
* Self-reported ULT adherence by Voils et al./3, 6, 9, and 12 months*	A validated questionnaire [22] with 2 scales, measuring the extent of non-adherence (3 items) and the reasons for non-adherence (21 items)	No defined threshold

*Analyses of 12-month MEMSCAP™ will indicate sustenance of the treatment effects noted at 6 months

**Table 2:** Schedule of visits and timing of each data point collection

	Baseline visit	Telephone visits (2 and 4 months)	3-month visit	6-month visit	9-month visit	12-month visit
MEMSCap™* adherence	**X**		**X**	**X**	**X**	**X**
**Secondary**						
*Number of gout flares*	**X**		**X**	**X**	**X**	**X**
*SATMED-Q*	**X**			**X**		**X**
*Serum urate*	**X**					**X**
*PEMAT-A/V*	**X**	**X**				
*GIS-GAQ*	**X**		**X**	**X**	**X**	**X**
*Self-reported ULT Adherence*	**X**		**X**	**X**	**X**	**X**

The 12-month visit (end-of-study) will be in-person, similar to the baseline visit, and include patient satisfaction with treatment and SU assessment. MEMSCap™ data will be downloaded, and veterans will complete a questionnaire including gout flares, GAQ, and self-reported medication adherence, as in 3-, 6- and 9-month follow-up. Patient satisfaction with treatment and SU will be done *only* at the 12-month follow-up to reduce patient burden (expected to take an additional 30-min). Veterans unable to come to the study clinic for the 12-month visit will be offered SU blood draw at their nearest community-based VA outpatient clinic (CBOC; a routine laboratory test), and the research associate will administer 12-month assessments via the phone interview.

The primary time point for analyses will be at 6 months. Assessments are kept short considering the responder burden and to ensure study retention. Veterans will be provided with a $25 remuneration for completing each study assessment; an additional $25 will be provided to those who perform both blood draws at baseline and 12-month end-of-study visits.

### Data management, quality assurance, and monitoring

Data management and quality assurance are particularly important for this study. All data collection will occur using HIPAA-compliant Microsoft Access® database or the VA Research Electronic Data Capture (REDCap; Nashville, TN) database on a secure VA server behind a firewall. Data entered by veterans at screening, baseline, and follow-up visits using the touchscreen computers will be directly captured in the study database. The study biostatistician will coordinate and oversee data management. Since personal identifiers will be collected, all database versions will be stored on the VA server. We will program logic and range checks in SAS 9.2 (SAS Cary, NC) to ensure timely identification of data fields requiring querying and clarification. A frequent multiple-backup strategy is proposed due to our desire not to lose any study data.

### Description of outcomes, outcome measures, and covariates (SA1 and SA2)

#### Choice of outcomes

All proposed primary and secondary study outcomes are clinically meaningful and patient-centered (Table 1). The primary outcome is ULT adherence at 6 months measured by MEMSCap™. Secondary outcomes are gout flares, patient satisfaction with treatment, SU, gout-specific health-related quality of life (HRQOL), and patient education materials assessment.

#### Primary outcome (SA1)

##### ULT adherence

We will calculate 3-, 6-, 9-, and 12-month ULT adherence using the MEMSCap™. MEMSCap™ is more accurate and has a higher validity compared to other measures of adherence (self-report, claims, etc.) [25], with excellent internal reliability (Cronbach’s alpha = 0.94),[32] high degree of agreement with pill counts (kappa = 0.72) [32, 33], and high predictive validity, given the association with lower symptom severity [32]. Despite some limitations, it is considered the best objective measure of medication-taking behavior. MEMSCap™ is child-resistant and wirelessly transfers the dosing data when used in conjunction with a MEMSCap™ reader. It can record and store up to 3800 dosing events. The mean ULT adherence at the 6-month period will be calculated. After careful consideration, we decided not to include probenecid, since it is given twice daily and constitutes only 5% of all ULTs prescribed. Allopurinol and febuxostat, ULTs taken once daily, will be included in this study.

#### Secondary outcomes (SA2)

##### Patient-reported gout flares

This will be assessed by the self-reported total number of gout flares in the last 1 and 2 months.

A validated gout flare will be assessed for patient-reported gout flare, based on the combination of a patient report of a gout flare along with the presence of any patient-reported warm joint, any patient-reported swollen joint, and patient-reported pain at rest score of > 3 (0–10 scale), a flare definition developed and validated [26]. This takes < 5 min to complete.

##### Patient satisfaction with treatment

Patient satisfaction will be assessed by the Satisfaction with Medications Questionnaire (SATMED-Q) [27]. It is designed for use with patients presenting with any chronic illness and taking any type of prolonged pharmacological treatment. SATMED has 17 items with six dimensions: treatment effectiveness, convenience of use, impact on daily activities, medical care, global satisfaction, and side effects adapted for our study. Responses are scored on a Likert scale from 0 to 4, as follows: 0 = “no, not at all”; 1 = “a little bit”; 2 = “neither a lot, nor a little”; 3 = “quite a lot”; and 4 = “yes, very much.” The total score ranges from 0 to 68, transformed to a 0–100 scale for ease of understanding. It takes < 5 min to complete. Thresholds for minimal clinical significant effect are shown in the table, i.e., for the total score, they were 5.9 to 13.4 points, and for the SATMED-Q domain, scores were 5.9 to 20.6, with most estimates close to 10 [29].

##### Serum urate

Serum urate will be determined by an enzymatic uricase method manufactured by Stanbio Laboratory (Boerne, TX), a standardized assay [29]. This biochemical outcome is a surrogate for disease control and the key target of ULT used by regulatory authorities for gout drug approval. Achieving and maintaining serum urate < 6 mg/dl (“target”) is associated with a lower risk of gout flares, tophi, and medical care costs [34–36], outcomes that are relevant to patients and providers, as well as the health care system.

##### Self-reported ULT adherence by Voils et al.

Self-reported medication adherence will be assessed using a validated questionnaire [22]. It has two scales, one measuring the extent of non-adherence (3 items scored from strongly disagree (score = 1) to strongly agree (score = 5) to produce an aggregate score (higher scores indicate greater levels of non-adherence)) and the other measuring the reasons for non-adherence (21 items scored from not at all (score = 1) to very much (score = 5; no total score calculated)). Intraclass correlations were 0.58 for the extent score and ranged from 0.07 to 0.64 for the reasons. It takes < 5 min to complete.

##### Gout-specific health-related quality of life (HRQOL)

Gout-specific health-related quality of life (HRQOL) will be assessed with the Gout Impact Scale (GIS) of the Gout Assessment Questionnaire (GAQ), a validated measure of the specific impact of gout on HRQOL [23]. GIS contains 22 questions (0–100 scale) that constitute 5 subscales. The clinically important difference of the GIS is between 5 and 8 points [31]. It takes 5 min to complete and will be done at 0 and 12 months.

##### Patient Education Materials Assessment Tool for Audiovisual Materials (PEMAT–A/V)

Patient Education Materials Assessment Tool for Audiovisual Materials (PEMAT–A/V) will be used to assess the understandability, accountability, and potential impact of various messages on change in behavior, including ULT adherence [30]; 16 items to assess understandability and 4 items for accountability are scored as agree = 1 and disagree = 0, with options for not applicable for some. We have adapted the scale to explore which material (stories vs. didactic) likely impacted behavior and which behavior changed because of the intervention. It will be done at 0 and 6 months.

#### Covariates

The following covariates will be assessed: (1) demographics—age, sex, marital status, and income; (2) gout duration and baseline gout flares; (3) body mass index (BMI; VA database); (4) alcohol use; and (5) baseline ULT MPR and baseline SU. We will analytically adjust for these covariates if they are not balanced by randomization**.**

### Statistical methods

For both aims 1 and 2, descriptive statistics for demographics (age, income, marital status, gout disease duration) and clinical parameters (ULT adherence, # gout flares, satisfaction, serum urate, GAQ) will be calculated. Specifically, central tendency measures (sample mean/median for continuous measures, proportions for categorical measures), dispersion measures (variance, range), and precision (95% confidence intervals) will be calculated. Given the two-arm study design (storytelling vs. usual care), statistical procedures appropriate for two-group comparisons (two-sample *t*-tests, tests of proportions, ordinary least square regression, logistic regression, Poisson regression methodology) will be utilized to conduct crude as well as adjusted comparisons based upon the distributional nature of the outcome. For ULT adherence, an unadjusted analysis will be conducted using the two-sample *t*-test. To control for age, sex, income, alcohol use, and other covariates, ordinary least squares regression will be used to test for treatment differences after adjusting for covariates. If the normality assumption is violated, nonparametric methods will be used, instead of the parametric tests. Similar approaches will be used to test for differences in patient satisfaction and HRQOL.

Poisson or quasi-Poisson regression will be used to test for group differences in gout flare rates in the last 1 or 2 months, while adjusting for covariates. Careful attention will be paid to the distributional assumptions for Poisson regression, and methods to adjust for overdispersion will be employed. Finally, separate logistic regression models will be used to measure treatment differences in the odds of achieving target serum urate < 6 mg/dl. To analyze the longitudinal data (3, 6, 9, and 12 months), we will use mixed linear models for ULT adherence (continuous outcome) and generalized estimating equations for target serum urate < 6 mg/dl (categorical). All analyses will be guided by intent-to-treat analysis principles.

No interim analyses were planned.

### Sample size and power

#### Hypothesis 1: We hypothesize that the mean ULT adherence will be higher in the intervention versus the comparison groups

Assuming a standard deviation (SD) of 15%, similar to the SD of 14% reported by Briesacher et al. [2], 120 patients/group (total of 306 to account for 18% drop out rate) will provide 80% power to detect an absolute difference between means of 6% for MEMSCap™ ULT MPR, assuming a control group adherence of 55%, intervention group mean of 61% (range, 0–100%) [2], and using a two-tailed type I error rate of 0.05. A difference of 6% equates to a medium Cohen’s effect size of 0.40. For a larger SD of 20%, we still have the power to detect between-group differences of 8% in MPR, which equals medium Cohen’s effect size of 0.53.

### Project management plan

#### Organizational structure

The STRIDE-GO Coordinating Center will be at the BHM VAMC under the direction of Dr. Singh. The Coordinating Center will provide scientific and administrative coordination of the study. This includes the development of the study protocol, scheduling of meetings and calls, answering questions about the protocol, site visits, progress reports, and administration of funds.

#### Performance standards and participant accrual requirements

The Coordinating Center will set performance standards and monitor site activities to assure recruitment and retention rates, and data quality and protocol adherence standards are met. Under-performing sites and issues will be identified, and solutions recommended. Periodic conference calls will help in resolving issues through the process of sharing experiences.

#### Adherence to protocol

Strict adherence to the protocol is mandatory throughout the course of the study; any anticipated deviation from it should be discussed prospectively with the Coordinating Center. The Coordinating Center will monitor the number of approved and unapproved protocol exceptions at each site and report these in interim statistical reports. Serious protocol violations include failure to obtain a valid informed consent and erroneously withdrawing participants from the study.

#### Data quality and audits

Continuous monitoring of study data quality will be jointly performed by the Coordinating Center biostatistician, as completed de-identified data are transmitted from participating sites. The monitoring will include the frequency of data problems such as missing data, unusual values, and inconsistent data. The Coordinating Center will review data as they are received for accuracy, completeness, consistency between related data items, and adherence to the protocol. Data query reports will be sent to the participating sites for processing. Data quality summaries will be generated monthly by the Coordinating Center and reviewed. The Coordinating Center will communicate commonly occurring problems to all sites and work directly with specific sites where higher error rates are detected. The Coordinating Center will perform data audits if the data quality summary indicates data issues. These audits will follow standards used in clinical trials, including the comparison of case report forms with primary data sources.

#### Timeline

Aims 1 and 2 will be achieved in 0–45 months and dissemination and implementation in 43–48 months (Table 3). This table also shows a plan for patient enrollment, follow-up assessments, and the study completion visit.

**Table 3:**
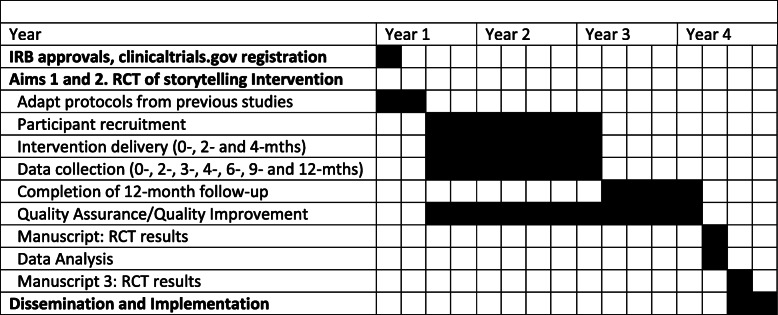
Study timeline

### Protocol modifications

In this study, we made two protocol modifications, both prior to study initiation. First, we found inconsistency between pharmacy-based vs. patient self-reported ULT MPR use during screening. We also found that there was a discrepancy for baseline ULT MPR < 80% determination using pharmacy record-based ULT MPR, based on the period selected, 3 vs. 6 vs. 12 months. We determined that pharmacy records were an imperfect measure and differed from MEMSCap™, our primary outcome measure. Therefore, we added a 1-month run-in period using MEMSCap™ prior to randomization and made this the measure of baseline ULT MPR rather than the pharmacy records of ULT prescription fill. Second, we found that many patients had allopurinol ULT MPR of 80% or higher during the 1-month run-in period. This indicated a possible Hawthorne effect of using MEMSCap™ and study participation on ULT adherence that would result in the potential exclusion of at-risk patients. Therefore, we changed the study inclusion criteria to allow the enrollment of eligible subjects regardless of their 1-month run-in period ULT MPR value, and pre-specified that in addition to the main analysis, we would also perform an analysis of all study outcomes, in particular primary outcome, by baseline ULT MPR of < 80% vs. higher. Other protocol modifications were related to the correction of typographic errors and for clarifications on the protocol for the site coordinators and site principal investigators.

## Discussion

ULT adherence in gout is low [16, 37, 38]. Therefore, effective interventions are needed to address this critical gap in gout care. We chose gout as a model of chronic disease with gout flares to test the efficacy of storytelling intervention for several reasons: (1) gout has a clear pathophysiology, and clinical features are linked to SU, which is the key abnormality; (2) the gold standard surrogate for disease outcomes in gout, SU, is primarily affected by ULT medication adherence [15, 16]; and (3) in contrast to gout, COPD and CHF have complex physiochemical mechanisms of dysfunction, have multiple causes for exacerbations (e.g., seasonal variation, infection), and have multiple approaches to treatment (drug class, routes).

A key reason to conduct this study in AAs with gout was the relative lack of observational data in minorities with gout and an absolute lack of data on data from trials on this subgroup of patients. The burden of gout is higher in AAs compared to Whites [3, 8, 9]. We recognized that enrollment of AA racial minority with gout in a clinical trial would be challenging, given the history of research in minorities in the USA, the widely prevalent distrust of research in the AA community, and the physical proximity of Birmingham, Alabama to Tuskegee, which is where the infamous Tuskegee study was conducted [39, 40]. However, the lack of effective interventions for AAs with gout in contrast to a disproportionate burden of gout in AAs was the key motivations. Therefore, we first developed a culturally appropriate gout-storytelling intervention for AAs with gout and designed this RCT for AAs with gout.

A successful trial completion will establish several important milestones for disparity research in rheumatic diseases: (1) culturally appropriate disease-specific patient-centered feasible behavioral interventions can be successfully developed for the AA minority population, based on qualitative work in the target population [41]; (2) high-quality RCTs can be conducted in AAs with rheumatic diseases; and (3) if the trial result is positive, an effective behavioral intervention for improving ULT MPR will be available for AAs with gout. Even if the storytelling intervention does not improve ULT adherence, the technique may be usefully applied to model other patient behaviors, such as patient-provider communication, appointment keeping, and lifestyle changes.

Limitations of the study include the lack of inclusion of economic evaluation in this trial and the lack of an independent steering committee. Strengths include a randomized controlled trial design, inclusion of attention control, a trial that will enroll African Americans with gout, test a culturally relevant and culturally appropriate intervention that will be easy to use in the future, if found to be effective.

## Supplementary Information

**Additional file 1.** Appendix 1

## Data Availability

The study PI and biostatistician will have access to the trial dataset. We are ready to share the data with colleagues, after obtaining appropriate permissions from the Veterans Affairs Ethics Committee, in accordance with the HIPAA and Privacy policies.

## Abbreviations

AA: African Americans
STRIDE-GO: SToRytelling to Improve DiseasE outcomes in Gout
ULT: Urate-lowering therapy
SU: Serum urate or serum uric acid
HBM: Health belief model
MEMS: Medication Event Monitoring System
MPR: Medication possession ratio
SATMED-Q: Satisfaction with Medications–Questionnaire
