# Supplementary material for: SToRytelliing to Improve Disease outcomes in Gout (STRIDE-GO) in African American veterans with gout: a trial study protocol
Source: Trials. 2021 Dec 4;22:879. doi: 10.1186/s13063-021-05847-9 (PMC8645140; doi:10.1186/s13063-021-05847-9)
Supplement: Supplementary file 1 — Additional file 1. Appendix 1 [file 13063_2021_5847_MOESM1_ESM.pdf]

Department of Veterans Affairs

VA RESEARCH CONSENT FORM

Subject Name ______________________________ **Date** ______________

Title of Study **SToRytelling to Improve DiseasE outcomes in GOut: The STRIDE-GO 2 study**

Principal Investigator **Jasvinder Singh, MD** **VAMC** **Birmingham (521)**

Sponsor

This research is supported by a grant from the VA. Study expenses will be paid for with money provided by the VA Office of Research and Development. All funds remaining after the completion of the study will be used for research purposes.

Introduction and Purpose

You are being asked to participate in this research because you are an African-American Veteran with gout and we value discussion of your experience with gout and gout medications. Gout is the most common inflammatory arthritis that affects 4% of U.S. adults, and 5% of Veterans. It is more common in African-Americans who have worse outcomes as compared to Caucasians. Due to the infrequent or occasional characteristic nature of gout, patients often don't understand its severity and vulnerability to disease complications, and, therefore, may not balance perceived barriers and benefits to treatment, which potentially contributes to low medication adherence and poor outcomes. The purpose of this study is to test a patient-centered, culturally relevant, narrative intervention or "storytelling" to improve patient outcomes in chronic diseases among African-Americans. Storytelling in patients' own voice has the power to directly and more effectively confront a patient's barriers to disease management and provides useful cues to action. Storytelling has been found to be a successful behavioral intervention for improving patient engagement and reinforcement by peer experience.

We will recruit 150 Veterans at the Birmingham VA Medical Center (BVAMC) for this study. Another 150 Veterans will be recruited from each of the other two sites: The Philadelphia and St. Louis VA Medical Centers. The study involves an approximate 2-hour visit which will include 1 interview about your gout and its treatment before and after watching a video on an iPad. We will invite you for more such visits at 3-month intervals for the next 12 months. In addition, a portable DVD player (if needed), with videos, will be mailed to you.

Procedures

Run-in period: The first 12-weeks of the study is called a run-in period. During the run–in period, you will be given your medication in a special bottle to understand how often you are able to take your gout medication. You will spend <30-minutes reviewing the consent form for the study. If you consent to be in the study, you will receive your gout medication in a bottle with special medication bottle cap that can count the number of times the bottle is opened. We will see you in the clinic anytime during these 12-weeks per your preference for 10-15 minutes to find out if you meet the criteria to be in the study. If you meet the criteria, we will proceed to enroll you in the study at this second visit. We will also consider you for the study in the future, but will not enroll you in the study.

VA - IR :
Consent Form Approved 7/16/18

Study period: We have produced a video for patients with gout made by patients with gout using storytelling with content uniquely tailored to African-American Veterans with gout, taking into account the cultural differences within them to help change behaviors to improve gout self-management. In order to help us test the video, you may participate in one or more of the following activities:

1. You will participate in the study in one of the two participant groups in the study. We will begin with a small survey that will focus on the barriers and challenges faced by you to regularly take your prescribed medications for gout. If you are in the first group, after the short survey, we will show you a video of other gout patients' stories on an iPad, which will be followed by another survey about your gout. This will help us understand how the video affects the understanding you have about your gout, any challenge you face and any solutions to those challenges you have found with regards to regularly taking gout medications.

   If you are in the second group, after the short survey, we will show you a similar video of information about a disease other than gout on an iPad, which will be followed by another survey about your gout. This will help us understand how the video message effects the understanding you have about your gout. In addition, you will be provided with special medication bottle caps to use for your gout medication so that we can learn about your medication use. We will request you to bring these caps with you on every visit. In addition, we will also collect your serum urate value done on the day of the first and 12-month study visit as a part of your routine care of your gout.

2. At around 2 months into your participation in the study, we will send you some DVDs with videos about gout by mail. Within a few days of sending these videos, we will call you on the phone for a short survey. This process will be repeated again at 4 months from the first study visit.

Risks and Discomforts

You may be inconvenienced and feel uncomfortable about answering some of the questions regarding your medical condition. You can choose not to answer those questions. Anxiety from responding to the questions is an anticipated risk to participating in the study. Assuring participant confidentiality is an essential component of this study. The information obtained during this study is confidential, and disclosure to third parties is prohibited.

Benefits
There could be direct benefits to you from this study. The study video might benefit you in that you may decide to take your gout medications more regularly and gain more knowledge about your gout and manage it better. Also, findings from this research may benefit other Veterans with gout in the future.

Alternative Treatment
Participation in this study is entirely voluntary and the alternative is to not participate.

Compensation/Payments
You will receive a VISA gift card or VA Form 10-7078 (Participant payment voucher) worth $25 for your participation for each session and for completing and sending each survey by mail. An additional $25 will be provided as VISA gift card or VA Form 10-7078 - Participant payment voucher to those who perform both blood draws at baseline and 12-month end-of-study visits. If payment voucher is used, participant will bring this voucher to agent cashier to receive $25 on the same day. A maximum total compensation of $200 is possible over the course of the study. All participants will retain the DVD player and DVDs after participating in the study.

Cost of Participation
Some Veterans are required to pay co-payments for medical care and services provided by VA. These co-payment requirements will continue to apply to medical care and services that are not part of this study.

Research-Related Injury
You will be participating in a research project approved by the BVAMC Research and Development Committee and conducted under the supervision of one or more VA employees. If you are injured as a result of your participation as a research subject in this study, the BVAMC will provide you with necessary medical treatment in accordance with Federal regulations. VA will not necessarily be responsible for treatment of injuries that result from noncompliance with study procedures, although veterans injured as a result of such participation may be eligible for care from VA under other statutory and regulatory provisions.

Any cost of care will be in accordance with your eligibility for care at VA. Care outside VA may not be free and VA may not pay for that care. If you have any questions regarding this study or you are injured and become ill as a result of participation in this study please call Dr. Jasvinder Singh at (205) 975-1758. If you need immediate medical assistance for a research-related injury, please call the BVAMC Emergency Room at (205) 558-4725.

Confidentiality

The research team will treat your identity with professional standards of confidentiality. The information obtained in this study may be published, but your identity will not be revealed. VA personnel, the BVAMC Institutional Review Board and other Federal oversight agencies reserve the right to inspect both the research data and your medical records. Absolute confidentiality cannot be guaranteed in all circumstances.

Voluntary Participation and Withdrawal

Participation in this study is voluntary, and you may refuse to participate without penalty or loss of benefits to which you are otherwise entitled. You are free to withdraw your consent and discontinue participation at any time. If you decide to withdraw from this study, the research team will stop using any further health information obtained from you. The research team will continue to use any information that they have already collected to ensure the integrity of the research. No new information will be collected. If you decide to withdraw from this study, you are asked to contact the Principal **Investigator, Dr. Jasvinder Singh, MD, at (205) 975-1758**. Discontinuation will in no way affect or jeopardize the quality of care you receive, now or in the future, at the BVAMC or your opportunity to participate in other studies. Your doctor may withdraw you without your consent, if you are unable to participate for medical reasons or if the study ends early.

New Findings

Any significant new findings that develop during the course of the research study that in the opinion of the investigator may affect your willingness to continue to participate will be provided to you as soon as possible.

Questions

For questions regarding the study, contact the Principal Investigator, **Dr. Jasvinder Singh, MD, at (205) 975-1758.** If you have any questions about the legitimacy of this study, your rights as a research participant, complaints/concerns about this research, or wish to discuss problems, obtain information or offer input, contact the Research Service Office at (205) 558-4747, and their staff will direct you to the appropriate person who can discuss your issue.

Department of Veterans Affairs

VA RESEARCH CONSENT FORM

Subject Name ______________________________ **Date** ____________

Title of Study **SToRytelling to Improve DiseasE outcomes in GOut: The STRIDE-GO 2 study**

Principal Investigator **Jasvinder Singh, MD** **VAMC** **Birmingham (521)**

You have read or have had read to you all of the above. Dr. Jasvinder Singh or a qualified designee from his research team has explained the study to you and answered all of your questions. You have been told about the risks, discomforts and possible benefits of the study.

You understand that you do not have to take part in this study. Your refusal to participate will involve no penalty or loss of rights to which you are entitled. You may withdraw from this study at any time without penalty or loss of VA or other benefits to which you are entitled.

The results of this study may be published, but your records will not be revealed unless required by law.

In case there are medical problems or questions, you have been told you can call **Dr. Jasvinder Singh at (205) 975-1758** during the day and the **BVAMC Emergency Room at (205) 558-4725** after hours. If any medical problems occur in connection with this study the VA will provide emergency care in accordance with your eligibility.

You understand your rights as a research subject and you voluntarily consent to participation in this study. You understand what the study is about and how and why it is being done. You will receive a signed copy of this consent form. **Please keep this form because it contains important phone numbers and other information.**

By signing and dating this informed consent, you are not waiving any of your legal rights.

		/ /
Participant's Name (printed)	Participant's Signature	Date (MM/DD/YYYY)
		/ /
Name of person conducting consent discussion (printed)	Signature of person conducting informed consent discussion	Date (MM/DD/YYYY)

ONLY WHEN APPLICABLE:

		/ /
*Name of Subject's Representative (printed)	Subject's Representative Signature	Date (MM/DD/YYYY)

*Only required if subject is incompetent

ICD Version Template Date: 7/11/14
VA Form 10-1086

VA - IRB
Consent Form Approved 7/16/18
